# Acupuncture Induces Reduction in Limbic-Cortical Feedback of a Neuralgia Rat Model: A Dynamic Causal Modeling Study

**DOI:** 10.1155/2020/5052840

**Published:** 2020-02-01

**Authors:** Zhen-Zhen Ma, Ye-Chen Lu, Jia-Jia Wu, Xiang-Xin Xing, Xu-Yun Hua, Jian-Guang Xu

**Affiliations:** ^1^Center of Rehabilitation Medicine, Yueyang Hospital of Integrated Traditional Chinese and Western Medicine, Shanghai University of Traditional Chinese Medicine, Shanghai, China; ^2^School of Rehabilitation Science, Shanghai University of Traditional Chinese Medicine, Shanghai, China; ^3^Key Laboratory of Hand Reconstruction, Ministry of Health, Shanghai, China; ^4^Shanghai Key Laboratory of Peripheral Nerve and Microsurgery, Shanghai, China; ^5^Department of Trauma and Orthopedics, Yueyang Hospital of Integrated Traditional Chinese and Western Medicine, Shanghai University of Traditional Chinese Medicine, Shanghai, China; ^6^Department of Hand Surgery, Huashan Hospital, Fudan University, Shanghai, China

## Abstract

**Background:**

Neuropathic pain after brachial plexus avulsion remained prevalent and intractable currently. However, the neuroimaging study about neural mechanisms or etiology was limited and blurred.

**Objective:**

This study is aimed at investigating the effect of electroacupuncture on effective connectivity and neural response in corticolimbic circuitries during implicit processing of nociceptive stimulus in rats with brachial plexus pain.

**Methods:**

An fMRI scan was performed in a total of 16 rats with brachial plexus pain, which was equally distributed into the model group and the electroacupuncture group. The analysis of task-dependent data determined pain-related activation in each group. Based on those results, several regions including AMY, S1, and h were recruited as ROI in dynamic causal modeling (DCM) analysis comparing evidence for different neuronal hypotheses describing the propagation of noxious stimuli in regions of interest and horizontal comparison of effective connections between the model and electroacupuncture groups.

**Results:**

In both groups, DCM revealed that noxious stimuli were most likely driven by the somatosensory cortex, with bidirectional propagation with the hypothalamus and amygdala and the interactions in them. Also, the 3-month intervention of acupuncture reduced effective connections of h-S1 and AMY-S1.

**Conclusions:**

We showed an evidence that a full connection model within the brain network of brachial plexus pain and electroacupuncture intervention reduces effective connectivity from h and AMY to S1. Our study for the first time explored the relationship of involved brain regions with dynamic causal modeling. It provided novel evidence for the feature of the organization of the cortical-limbic network and the alteration caused by acupuncture.

## 1. Introduction

Neuropathic pain (NP) after brachial plexus avulsion (BPA) constitutes a major mental health burden on patients who suffer from the lower ability of upper extremity in motor and sensory deficits physically. Neuralgia after brachial plexus injury (BPI) is not a single problem of peripheral nerve injury, which may share the same etiology as phantom limb pain [1, 2]. Multiple reports indicate even amputees would not be helpful in pain relief for BPI patients [3, 4]. These facts lead to an understanding that neuralgia after BPI is closely associated with self-cognition or brain plasticity [5, 6]. Researches on brain plasticity provide novel information about the origin of BPI neuralgia. And thus, it would contribute to the development of a new treatment strategy.

Previous research gradually managed to identify the brain regions functionally corresponding to pain syndrome [7, 8]. Several specific regions including the cingulate cortex, thalamus, insula, prefrontal cortex, inferior parietal cortex, and primary and secondary somatosensory cortices were found to share the same pattern of activation confronting noxious stimulus, which were reported as “pain matrix” [9, 10]. Such knowledge about pain-related brain regions remained robust in multiple researches about neuralgias as revealed by recent works [11–13]. However, it is still difficult to interpret the entire mechanism of neuralgia based on the activation of brain regions in the pain matrix. Currently, an increasing number of researches have switched their focus from mapping single activation regions towards describing the correlation between brain regions [14, 15]. However, few research provides any explicit information about how the distributed activity is caused and propagates within a network [16].

Our preliminary studies showed that electroacupuncture affected peripheral nerve injury manifested as a synchronized activation pattern in the somatosensory and pain-related areas and limbic/paralimbic system. Specifically, the somatosensory area and pain-related areas were overactivated after modeling and then suppressed when electroacupuncture was applied. The limbic/paralimbic areas much more fluctuated during the electroacupuncture intervention [17, 18]. Therefore, we would select critical pain-related regions under task-dependent circumstances and build a directional network using a model language to study the initiation and propagation of pain circuity.

## 2. Materials and Methods

### 2.1. Animals

All protocols and procedures of animals followed the guidelines of the Biomedical Resource Center and were approved by the Institutional Animal Care and Use Committee. Model establishment damaging the right brachial plexus from C5 to T1 eliciting pain were conducted on 30 adult female Sprague-Dawley rats [19]. And we screened the rats based on the presence of self-harm behavior to distinguish which of them presented neuropathic pain syndrome. 16 out of 30 rats remained in the study after the selection, which were randomly divided into two groups equal in number: the model group and the electroacupuncture (EA) group.

### 2.2. Behavioral Assessment and Analysis (Mechanical Withdrawal Threshold (MWT))

Withdrawal response of the rats to mechanical and thermal stimuli was measured at the time of pre-modeling, 1-month postmodeling, and after 3-month treatment. We used a series of ascending force von Frey monofilaments (Stoelting, IL) to measure mechanical sensitivity on both hindpaws. The threshold of MWT was taken as the lowest force that evoked a brisk withdrawal response, at least two out of four times repetitive stimuli [19]. The MWT results were analyzed by nonparametric statistical analysis (*P* < 0.05) using the statistical package SPSS 20.0.

### 2.3. Acupuncture Intervention

The acupoints “Jiaji” (EX-B2) were chosen for EA treatment [20]. Rats were kept without anesthesia in a self-made wooden compartment apparatus, with the back exposed. Three disposable stainless-steel needles (0.25 × 13 mm, Huatuo acupuncture needle) were inserted into the contralateral Jiaji points to the injury along the C5-C7 levels. The top and down two needles were connected to the output terminal of the stimulator and stimulated dense disperse waves of 2/15 Hz frequency and 0.2 mA intensity for 15 min [21]. Acupuncture intervention started 1 month after modeling and continued for 5 days, 5 times per week for 3 months.

### 2.4. fMRI Acquisition and Preprocessing

For each rat in the experiment, five minutes of fMRI recordings with a block design sequence was acquired on a 7 T (Bruker Corporation, Germany) MRI scanner. The parameter settings of blood oxygen level-dependent (BOLD) signal were referred to our prior work [19]. fMRI data were corrected for upscaling (magnification 10×), stripping none-brain tissue, reorientation, slice-timing, realignment, coregistration, spatially warped and resampled to the template in MNI space, and spatially smoothed with an isotropic Gaussian kernel (full width at half maximum) twice as the voxel size.

### 2.5. Whole-Brain Analysis

The first-level analysis was conducted for each rat by building a general linear model (GLM), using the individual onset and duration of each trial for the stimuli task. The head-motion parameter derived from realignment procedure was regressed out in the model. One sample *t*-test on the contrast image for the canonical stimuli regressor was used to obtain statistical parametric maps of task-related BOLD signal changes. The threshold was set at a voxel level, 2-tailed *p* < 0.01. Then, the second-level analysis of two-sample *t*-test was adopted to identify the difference of sensory stimulus-response between the EA and model groups at three-time points. Significant thresholds were set at *p* < 0.05. And penetrance maps were calculated for each comparison to convey the spatial consistency of BOLD signal change across the rat groups.

### 2.6. Dynamic Causal Modeling (DCM)

DCM was used to select the most plausible combinations of intrinsic connections and driving inputs from the given data through Bayesian model selection [22]. The fMRI analyses described above identified active brain regions during right forelimb stimuli, but they did not permit the inference of causal relationships. DCM was therefore used to compare competing models of how interactions may have generated from our data. Analyses were performed with the DCM module in SPM, with models parameterized using the bilinear differential equation.

#### 2.6.1. Region of Interest (ROI)

In our study, we focused on the analysis of the pain processing network model consisted of the somatosensory cortex, hypothalamus, and amygdala in the left hemisphere, based on the group-level task-dependent fMRI results and the following considerations: (1) the somatosensory cortex area as part of the sensory system that is direct and sensitive to pain stimuli [23], (2) the hypothalamus that is associated with pain regulation and involved in integrating affective and contextual information [24], and (3) the amygdala for its role in emotional-affective and cognitive effects of pain processing [25].

The selection and functional localization of our volumes of interest (VOI) were positioned within significant individual-level activation clusters in each group separately. Rats' individual spherical VOIs were centered on the peak voxel (radius 5 mm, *p* < 0.05) in the respective contrast, and the first eigenvariate was extracted as a summary statistic for all active voxels within the VOI.

#### 2.6.2. Model Specification

**Figure 1:**
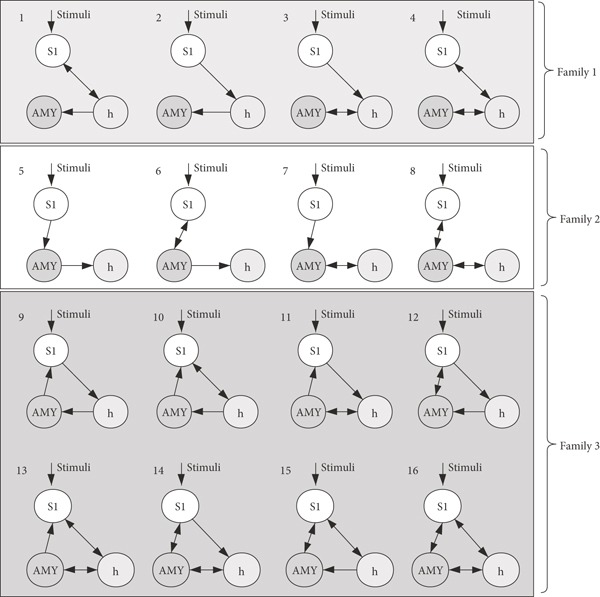
Framing of dynamic casual model (DCM) structures. 16 dynamic causal modeling structures were defined as the common stimuli (i.e., driving input) but with different effective connectivity (i.e., propagation pathway); therefore, they were divided into 3 families. 3 ROIs (i.e., ROI S1 = somatosensory cortex, ROI AMY = amygdala, and ROI h = hypothalamus) were specified in the models from the left brain.

Sixteen model structures were specified based on the considerations of group-level BOLD signals and neurophysiological prior knowledge. Each represented a different hypothesis concerning connections, here referred to as the signal pathway. Driving input was specified to originate at the somatosensory and here referred to the square waveforms representing stimuli onsets/durations [26–28]. Hence, we included models with all possible intrinsic connectivity patterns within the three regions in the model space. That means, we specified bidirectional (both forward and backward) or unidirectional (forward only) intrinsic connections for each interregional connection, yielding 16 specified and hypothetical models in all (Figure 1).

#### 2.6.3. Model Comparison

Bayesian model selection (BMS) uses the free energy, a lower-bound approximation to the log-model evidence, accounting for both model accuracy and model complexity [29, 30]. We used fixed-effects (FFX) BMS to compute the exceedance probabilities of model families within the given models for each group in two steps. First, models were sorted into three families based on the three propagation hypotheses, and the family-level inference was used to determine the family with the most likely propagation pathway. Finally, the remaining 8 models were compared using model-level inference to determine the most likely directionality of interregional connections. Bayes factors have been applied as grading criteria for the comparison of scientific theories. The most plausible family or model was identified as the one with the highest Bayes factors and exceedance probability [31].

#### 2.6.4. Effective Connection Comparison

Then, we extracted the parameters of the winning model to make further inferences, whether the connectivity in the model as specific couplings is modulated by the remodeling or treatment. Consequently, we conducted multiple two-way ANOVA grouped analysis of the connectivity parameters using GraphPad prism 7.0 and Turkey's multiple comparisons test to control the false discovery rate (*p* < 0.05).

## 3. Result

### 3.1. Group Differences of Behavior Assessment

**Figure 2:**
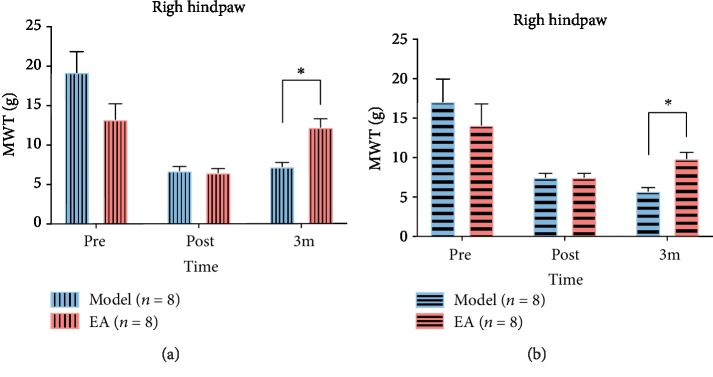
Result of mechanical withdraw threshold (MWT) between the model and EA groups. (a) Results of MWT between groups on the right hindpaw. (b) Results of MWT between groups on the left hindpaw. ^∗^*p* < 0.05 (at individual time points between the model and EA groups).

At the first observation point (presurgery) and the second observation point (post-surgery), the behavioral analysis between groups was insignificant (*p* = 0.108 (at a presurgery time point on the right hindpaw), *p* = 0.733 (at a presurgery time point on the left hindpaw, *p* = 0.317 (at a post-surgery time point on the affected hindpaw), and *p* = 1 (at a post-surgery time point on the unaffected hindpaw)). Only at the time point of after 3-month treatment, MWT between groups on the bilateral hindpaws was shown significant (*p* = 0.017 (affected hindpaw), *p* = 0.011 (unaffected hindpaw)), that is, the neuropathic pain caused a decrease in the MWT after modeling and an increase after 3 months of treatment with EA (Figure 2).

### 3.2. Group Differences of Activation in Block-Design Scan

**Figure 3:**
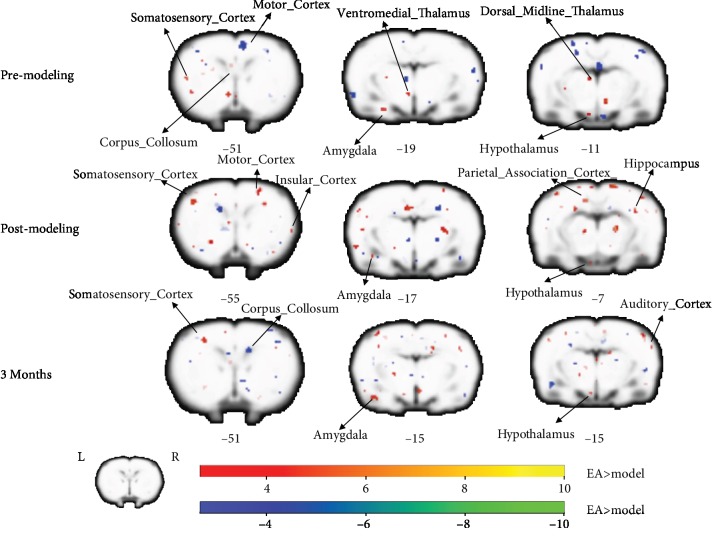
Group differences of activation in block-design scan. The three rows displayed the results of the group difference in right-forelimb stimulation task at the three time points, respectively (i.e., premodeling, postmodeling, after 3 months of treatment). The warm tone represented greater activation of the EA group than that of the model group, whereas the cold tone represented weaker activation of the EA group than the model group. The *Z*-value was the *z*-axis coordinate along the anterior-posterior axis referenced to a stereotaxic rat brain MRI template which has been aligned with the coordinates of Paxinos and Watson's.

Across all 16 rats, a total of 10 stimuli events recorded in scanner were analyzed. From the analysis of right-limb stimulation task, the statistical analysis between the two groups showed a similar pattern that extensive areas of the frontal, parietal, temporal, cingulate, and insular cortices, and the limbic system were detected to have significant BOLD signal changes accounted for the task at different time points. Especially, BOLD signal increases seen in the EA group after 3 months of treatment were the contralateral motor cortex, somatosensory cortex, and superior colliculus, and ipsilateral dorsal midline thalamus, somatosensory cortex, and amygdala. BOLD signal decreases were noted in the contralateral putamen caudate, amygdala, piriform cortex, callosum corpus, and ipsilateral septum (Figure 3).

### 3.3. Family-Level and Model-Level Results

**Figure 4:**
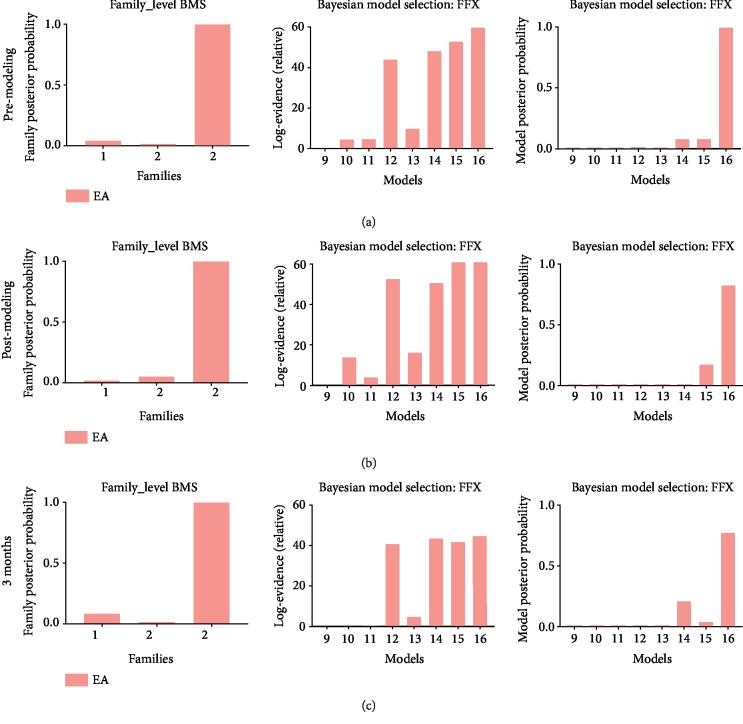
Results of Bayesian model selection for the EA group. Results of BMS for the EA group at premodeling (a), postmodeling (b), and after 3 months of treatment (c). The first column represented results of family-level inference in which sixteen models were sorted into three families based on the region with pain propagation pathways. The second column showed the Bayes factors to prove the evidence level of posterior probability. The third column displayed results of model-level inference in which the eight models from the winning family were compared to determine the most plausible directionality of interregional connections underlying pain propagation.

Figure 4 displayed the results of FFX BMS with family-level inference and model-level inference for the EA group at different time points (i.e., premodeling (a), postmodeling (b), and 3 months after treatment (c)). Family 3 in which the three regions merged a closed loop, with a posterior probability of 1, was most plausible. Model 16 was shown most fitting in the EA group (posterior probability of 0.999 at premodeling, 0.826 at postmodeling, and 0.773 at 3 months; Bayes factor of 60 at premodeling, 66.2 at postmodeling, and 44.8 at 3 months).

**Figure 5:**
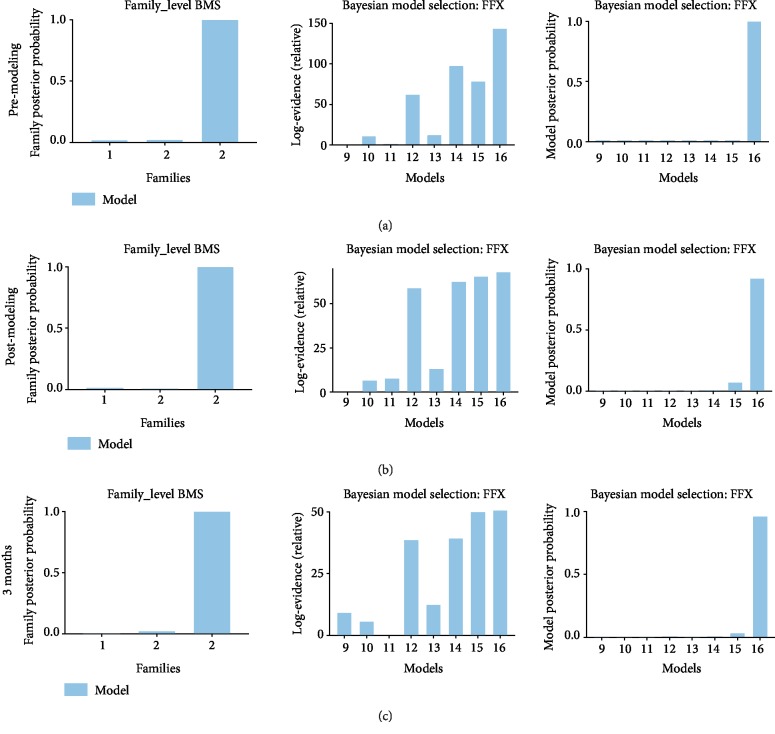
Results of Bayesian model selection for the model group. Results of BMS for the model group at premodeling (a), postmodeling (b), and after 3 months of treatment (c). The first column examined which interregional connections are most likely to mediate pain propagation; the second column showed the corresponding Bayes factor, in line with the results of model-level inference; and the third column displayed the results of model-level inference.

Figure 5 displayed the results of FFX BMS for the model group at different time points (i.e., premodeling (a), postmodeling (b), and 3 months after treatment (c)). Equivalently, family 3 with a posterior probability of 1 was most plausible; model 16 was demonstrated most proper in the model group (posterior probability of 0.999 at premodeling, 0.826 at postmodeling, and 0.773 at 3 months; Bayes factor of 60 at premodeling, 66.2 at postmodeling, and 44.8 at 3 months).

### 3.4. Effective Connection Results

**Figure 6:**
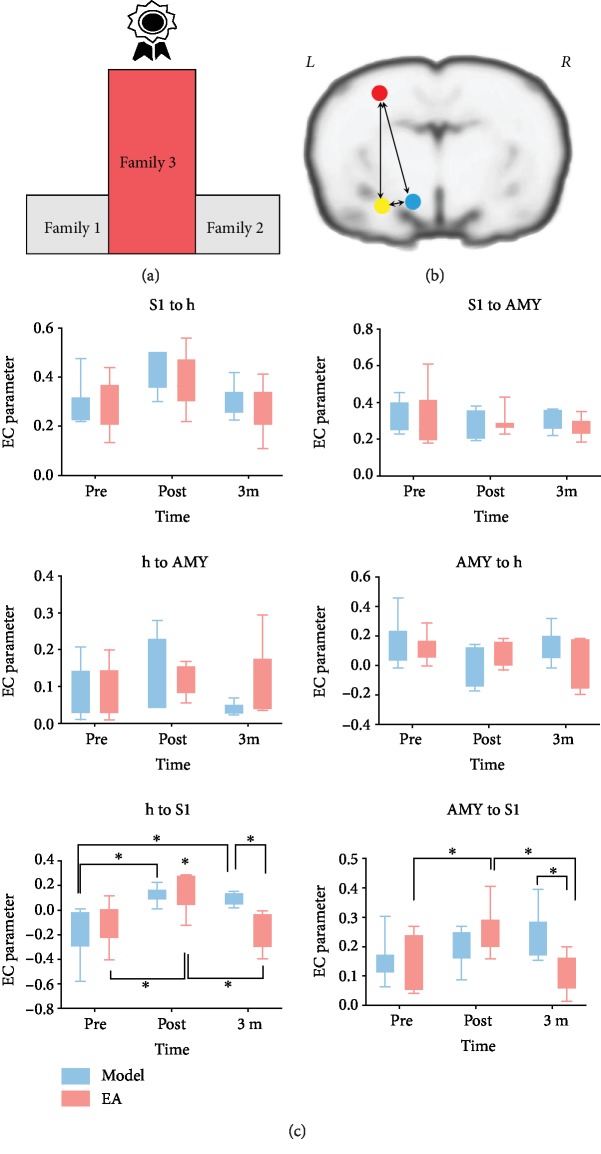
Group effective coupling differences of postulated cortically driven limbic network. (a) Schematic representation of the result of the winning family. (b) Schematic representation of interacting pain matrices. The red sphere represents the somatosensory cortex, the yellow one is the amygdala, and the blue one means the hypothalamus. (c) Results of group analysis of the effective connectivity between the model and EA groups. Each mini-graph represents the result of each connection in the winning model (i.e., h-S1, AMY-S1, S1-h, S1-AMY, h-AMY, and AMY-h).

The timing of these variables may be particularly critical and need to be studied in longitudinal investigations. The analysis shows that the coupling from h to S1 was significant between premodeling and postmodeling in both groups (adjusted *p* value is 0.0014 in the model group and is 0.0084 in the EA group), postmodeling and 3 months treatment in EA group (adjusted *p* value is 0.0025). Additionally, it shows the coupling from AMY to S1 was significant within the three-time points in the EA group (premodeling vs postmodeling: *p* = 0.0384, postmodeling vs 3-month intervention: *p* = 0.0072) (Figure 6). While the cross-sectional research is needed to clarify the potential role of EA, only the intrinsic couplings from h to S1 and AMY to S1 couplings between the two groups after 3 months of intervention are significant (premodeling vs postmodeling: *p* = 0.0232, postmodeling vs 3-month intervention: *p* = 0.0127) (Figure 6(c)).

## 4. Discussion

Our study examined the noxious stimuli-dependent functional architecture of the cortical-limbic system network during pain processing using DCM. Specifically, we used a dynamic task-related paradigm to assess activity and connectivity in brain regions supporting pain processing and, subsequently, whether and which electroacupuncture modulates effective connectivity of the network. The results of our research suggested three main conclusions. Firstly, we corroborated earlier studies by showing that the somatosensory cortex as a direct response region of somatic regulation is especially sensitive to noxious stimuli. Our data suggested that during the processing of pain, the limbic system including the amygdala and hypothalamus was strongly activated. Secondly, the model comparing results directly demonstrated that activity in key regions of the cortical-limbic network during pain processing was best explained by bidirectional contextual modulation of effective connectivity. Accordingly, processing pain directly induced changes in the coupling strengths within the cortical-limbic circuitry. Thirdly, we found evidence for a differential effect of modulation on the coupling between regions of AMY and S1, and h and S1. This suggested that the limbic system not only served the integration of receiving the signals from S1 but also continuously exerted influence on S1 during treatment of electroacupuncture.

The active regions with brachial plexus pain spatially overlapped with brain areas implicated in cognitive impairment in neuropathic pain [32–35]. Pain was considered multidimensional and produced by distributed neural patterns [7, 36]. Previous studies have highlighted the role of pain matrix during the pain processing, and they have realized the several interacting networks activated by painful stimuli, namely, neurosignature patterns for pain [37]. Moreover, recent work proposed a three-level network evolved by the concept of pain matrix. The first-order processing is about a nociceptive cortical matrix, then from nociception to a second-order perceptual matrix, including posterior parietal, prefrontal, and anterior insular areas. Their joint activation is necessary for conscious perception, attentional modulation, and control of vegetative reactions. At last, the third-order networks, including the orbitofrontal and pregenual/limbic networks, can still be modified as a function of beliefs, emotions, and expectations [37].

Our data showed significantly increased activity and interrelation in the ROIs we selected, present in the model and EA groups at the modeling and treatment course. Our findings may, therefore, reflect that the pain encoding is most likely driven by the somatosensory cortex before propagating to the amygdala and hypothalamus. The cortical driver of activity in neuropathic pain had been identified in a previous study [38, 39]. Anatomically, the somatosensory cortex is well-positioned to process communication with spinal sensory neurons in response to sensory inputs [40]. As the nociceptive cortex, studies indicated that stimulus-evoked responses of S1 corticofugal neurons can contribute to somatosensory processing by modulating noxious inputs in the spinothalamic system [23].

The paradigm of noxious stimuli activated the hypothalamus region, an area that was included in the limbic system. The fact that the hypothalamus has numerous reciprocal connections with other limbic regions suggests that they might be critical to producing emotional responses to noxious stimuli. The activation in the hypothalamic neurons may also mediate the autonomic response to the traumatic painful experience [24]. The stress responses were mediated by largely overlapping circuits from spinal pathways to the limbic system and hypothalamic centers such as the neuroendocrine and autonomic systems [41]. Concerning the behavioral responses induced by stimulating the hypothalamus, numerous researches implied that the hypothalamus was responsible for the maintenance of attention/arousal and might mediate the connection between higher cognitive states and physiological responsivity [42–45].

Pain carries a negative affective valence and is considered closed to anxiety and depression [46–48]. The amygdala is now recognized as a key player in emotions and affective disorders of pain [45, 49, 50]. Substantial animal and human studies demonstrated that the amygdala modulates behavioral responses to noxious stimuli, serving as a neural center for the modulation of pain perception [51–54]. In the present study, we found that activated changes in the amygdala play a crucial role in modulating reaction associated with pain stimuli and we further identified the underlying mechanism of interactions with the somatosensory cortex and hypothalamus.

Given the distributed time series signals in S1, h, and AMY during pain processing, we tested how these brain regions are functionally coupled with each other to mediate signal-guided transmission. Accordingly, we employed DCM [26] for inferring the most likely generative model of effective connectivity. However, no matter which group or time point it was, the most plausible neuronal model in brachial plexus pain was identified as the full directional model which all related regions have an interrelationship with the other one.

Together, these data suggested that the interaction of ROI regions we selected may be responsible for signal exchange characteristics of neuropathic pain. Neuropathic pain was associated with changes in each of these levels of integration. All the regions among matrices that were involved in the processing of noxious signals permitted continuous information transfer and continuously reconstructed and created a dynamic pattern of interactions. The observed signal changes might have reflected task-dependent facilitation of, rather than spontaneous, response. This not only followed recent theories of distributed and collaborated processing of painful stimuli among regions [12, 55] but also provided direct evidence for the idea that the cortical-limbic circuitry changes its functional state to support appropriate mental functions for a given context.

Furthermore, we studied whether the electroacupuncture also influenced the effective couplings. Assuredly, we concentrated on the coupling between h and S1, and AMY and S1. All the sides existed naturally because of the intrinsic connections; however, the modeling and electroacupuncture made sense mainly on the coupling of h to S1 and AMY to S1. That said, cortical-limbic effective connectivity between h and S1, and AMY and S1 was significant during pain processing with management of acupuncture. Generally, the pain-sensitive coupling between limbic regions and the somatosensory cortex in our dynamic models could reflect a cognitive attenuation of painful stimuli, which would eventually yield an adaption of sensuous response mediated by the AMY and h. However, it might reflect a downregulation mechanism of electroacupuncture that is dampening the induced increasing connectivity to S1 by the limbic regions to painful stimuli cues. These results provided reproducible evidence of a shared interaction of information flow within the brain network of the pain pathway and highlighted potential mechanisms that electroacupuncture contributed to the way in the pain path and reversed the remodeling [56–58].

## 5. Conclusion

We concluded that acupuncture intervention reduced effective connectivity from h and AMY to SI. That could be one of the cortical mechanisms which result in the therapeutic effect of pain relief. Our study for the first time explored the relationship of involved brain regions with dynamic causal modeling. It provided novel evidence for the feature of organization of the cortical-limbic network and the alteration caused by acupuncture. Understanding mechanisms of dynamic integration of cognition changes in the pain processing network might be pivotal for the further interpretation of the etiology of neuralgia and the mechanism of acupuncture therapy.

## Data Availability

The data used to support the findings of this study are available from the corresponding author upon request.
